# Salvage anlotinib showed sustained efficacy in heavily pretreated EGFR wild-type lung adenocarcinoma: A case report and review of the literature

**DOI:** 10.1097/MD.0000000000022707

**Published:** 2020-10-09

**Authors:** Lei Liu, Xiang Wang, Wen-Bin Wu, Miao Zhang

**Affiliations:** a Institute of Digestive Disease, China Three Gorges University, Department of Gastroenterology of Yichang Central People's Hospital, Yichang, China; b Department of Thoracic Surgery, Xuzhou Central Hospital, Xuzhou, China.

**Keywords:** anlotinib, epidermal growth factor receptor (EGFR), tyrosine kinase inhibitor (TKI), wild-type

## Abstract

**Rationale:**

Anlotinib has been proved to be effective in advanced refractory non-small cell lung cancer.

**Patient concerns:**

A 47-year-old female non-smoker was admitted due to persistent chest tightness for a month.

**Diagnoses:**

Epidermal growth factor receptor (EGFR) wild-type advanced primary lung adenocarcinoma without brain or bone metastasis.

**Interventions:**

The patient failed 2 lines of pemetrexed/docetaxel plus carboplatin and third-line erlotinib. Fourth-line anlotinib was administered thereafter.

**Outcomes:**

The pulmonary lesions showed partial remission 5 months after anlotinib monotherapy. The patient demonstrated a progression-free survival of more than 7 months and an overall survival of >12 months. The adverse events including hypertension and fatigue were well-tolerated.

**Lessons:**

Salvage anlotinib might be a reasonable choice in EGFR wild-type lung adenocarcinoma after failure of chemotherapy. Further well-designed trials are warranted to verify this occasional finding.

## 1 Introduction

Anti-angiogenesis is a promising therapeutic approach against solid tumors. However, most small-molecule vascular endothelial growth factor receptor (VEGFR) tyrosine kinase inhibitors (TKIs) including sunitinib, sorafenib, and pazopanib have low selectivity.^[1]^ Anlotinib (AL3818) is a multitarget TKI,^[1]^ which has been approved as third-line treatment for refractory non-small cell lung cancer (NSCLC) in China since May 2018. The activity of anlotinib mainly depends on its highly specific suppression of VEGFR2.^[1]^ Anlotinib demonstrates a broad antitumor capacity with manageable adverse events (AEs).^[2]^ The recommended dosage of anlotinib is 12 mg daily on the 2-week on/1-week off schedule because the plasma concentration of anlotinib reaches its maximum on day 14 and decreases subsequently.^[3]^

The current evidence of anlotinib in epidermal growth factor receptor (EGFR) wild-type NSCLC is insufficient. Herein we presented a case of chemotherapy-refractory and EGFR wild-type lung adenocarcinoma that showed significant response to fourth-line anlotinib monotherapy. Meanwhile, the relevant reports and registered trials of anlotinib for the treatment of lung cancer were briefly reviewed.

## 2 Case presentation

A 47-year-old female nonsmoker was admitted in Oct. 2018 due to persistent chest tightness for a month. The physical examination showed right-sided diminished breath sounds, whereas the laboratory tests recorded elevated serum carcinoembryonic antigen (CEA) and cytokeratin-19 fragment (CYFRA 21-1). The chest x-ray indicated right-sided pleural effusion; therefore, chest tube drainage was performed. The cytological study of the pleural effusion revealed malignant cells. The other potential comorbidities such as congestive heart failure, hepatic cirrhosis and hypoproteinemia were excluded during the differential diagnosis.

**Figure 1:**
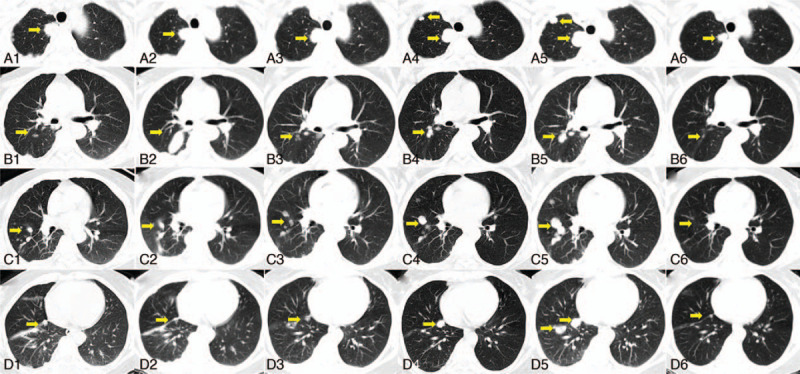
The pulmonary lesions during the treatment. (A1–D1): At least 4 measurable lesions (indicated by arrows) were shown before treatment in Oct. 2018. (A2–D2): Partial remission was observed after 1 cycle of first-line pemetrexed. (A3–D3): Progressed disease was shown after 4 cycles of pemetrexed. (A4–D4): Progressed disease of the lesions was recorded after 1 cycle of second-line docetaxel. (A5–D5): Progressed disease of the lesions was shown after 3 weeks of third-line erlotinib. (A6–D6): The lesions in the right middle/lower lobes and the right upper lobe showed complete remission and partial remission respectively 5 months after the fourth-line anlotinib monotherapy.

Further chest computed tomography (CT) showed extensive pleural and pulmonary nodules (Fig. 1A_1_–D_1_) and slightly enlarged mediastinal lymph nodes. Percutaneous lung biopsy confirmed the diagnosis of primary lung adenocarcinoma. In addition, EGFR mutation or anaplastic lymphoma kinase rearrangement was not identified by liquid biopsy using the pleural effusion and peripheral blood samples of the patient. Considering the potential risk of iatrogenic tumor dissemination, lymph node biopsy was avoided. Brain or bone metastasis was excluded by magnetic resonance imaging (MRI) and emission CT (ECT). Based on the above findings, the patient was staged as cT4NxM1a, IVA according to the 8th edition of TNM staging system.^[4]^

**Figure 2:**
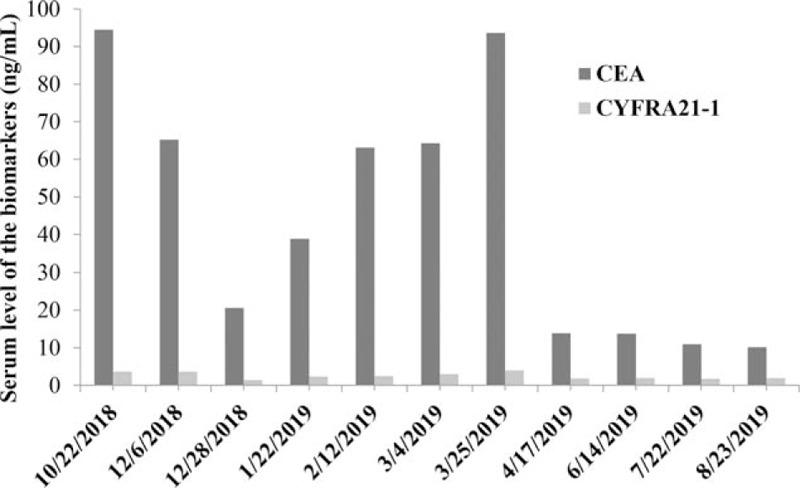
The changes of serum CEA and CYFRA21-1 during the therapy. CEA = carcinoembryonic antigen, CYFRA 21-1 = cytokeratin-19 fragment.

First-line pemetrexed (500 mg/m^2^ of body surface area) plus carboplatin (area under curve = 5) every 21 days was administered. The efficacy and toxicities were evaluated according to Response Evaluation Criteria in Solid Tumors (RECIST 1.1) and National Cancer Institute Common Terminology Criteria for Adverse Events version 4.0 respectively. Stable disease of the lung lesions was indicated after 1 cycle of pemetrexed in December 2018 (Fig. 1A_2_–D_2_); however, progressive disease (PD) was shown after 4 cycles of pemetrexed in February 2019 (Fig. 1A_3_–D_3_). In addition, the serum CEA was elevated since December 2018 (Fig. 2). Therefore, second-line docetaxel (75 mg/m^2^ of body surface area) plus carboplatin was provided. However, PD was also indicated after 1 cycle of docetaxel in March 2019 (Fig. 1A_4_–D_4_). The patient refused further intravenous treatment and took oral erlotinib (150 mg per day) as third-line therapy regardless of the wild-type EGFR status. Nevertheless, the patient was readmitted due to aggravated chest stiffness about 3 weeks later. An emergency chest x-ray showed PD of the lung lesions in March 2019 (Fig. 1A_5_–D_5_).

Although the immune checkpoint inhibitors could be considered next, they were not covered by her health insurance and the patient was not appropriate to be enrolled in the ongoing trials of immunotherapy according to their inclusion/exclusion criteria. After a multidisciplinary evaluation, fourth-line anlotinib as salvage treatment was administered (12 mg daily on day 1–14 followed by 7 days off) since March 2019. This regimen was assumed to be continued until PD or uncontrolled adverse events. Encouragingly, the serum CEA of the patient decreased obviously in April 2019 (Fig. 2). Five months after anlotinib treatment, the chest CT in August 2019 reported nearly a complete remission of the lung lesions in the right middle and lower lobes, followed by a partial remission of the lesion in the right upper lobe (Fig. 1A_6_–D_6_). Moreover, grade 2 hypertension and elevated serum aspartate aminotransferase were efficiently controlled. The patient therefore demonstrated a progression-free survival of >7 month, and an overall survival of >12 months. Further follow-up is continued. In detail, the serum CEA and CYFRA 21-1, cranial MRI, chest CT, and bone ECT were performed every 3 months.

## 3 Discussion

The present case indicated the potential benefit of anlotinib monotherapy in chemotherapy-refractory lung adenocarcinoma harboring wild-type EGFR status. The inhibition of autophagy potentiates the antiangiogenic property of anlotinib.^[5]^ In addition, anlotinib also displays antiproliferative and proapoptotic effects with a low incidence of grade ≥3 adverse events,^[6]^ and exhibits pharmacokinetic characteristics similar to sunitinib, sorafenib and nintedanib.^[7,8]^ Furthermore, there are about 127 genes that are associated with anlotinib resistance.^[9]^

Reliable biomarkers to evaluate the efficacy of anlotinib have not been fully elucidated yet.^[10]^ The CD31-labeled activated circulating endothelial cell is a potential indicator for predicting the efficiency of anlotinib in NSCLC, although it requires further validation.^[11]^ CEA of the present patient seems to be an informative indicator of anlotinib because the change of serum CEA was to some extent parallel with the radiographic changes of the pulmonary lesions. More reliable biomarkers such as circulating DNA which is capable of predicting and monitoring the response to anlotinib require further validation.^[12]^

We searched PubMed, Web of Science, Scopus, and Cochrane Library databases till February 2020 for published reports of anlotinib monotherapy in advanced refractory NSCLC. Finally, a total of 4 studies involving 453 patients were obtained and listed in Table 1.^[13–17]^ The trial ALTER0303 indicated a longer median overall survival (OS) and PFS of the NSCLC patients in the anlotinib group compared to those in the placebo group (9.6 months vs 6.3 months and 5.4 months vs 1.4 months, respectively).^[13]^ Meanwhile, anlotinib improved the quality of life of the patients as compared with placebo.^[13,17]^ The trial ALTER0302 also showed that the median PFS (4.8 vs 1.2 months) and objective response rate (10.0% vs 0%) were better in anlotinib than placebo in NSCLC patients who had not received immunotherapy.^[14]^ On the contrary, another trial (NCT02388919) reported that anlotinib did not provided a better OS versus placebo in chemotherapy-refractory NSCLC patients.^[15]^

**Table 1:**
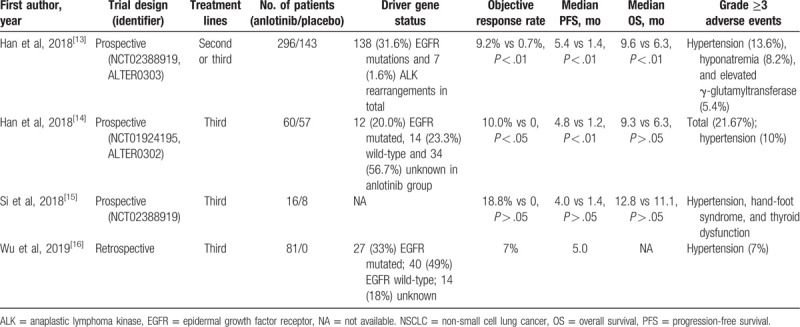
Previous reports of anlotinib monotherapy for advanced refractory NSCLC patients.

However, immunotherapy and antiangiogenic TKIs can also be considered for EGFR wild-type NSCLC after chemotherapy. About 10% of EGFR wild-type patients respond to TKI, but the benefit is modest with unknown mechanisms of sensitivity.^[18]^ It is noteworthy that there is little rationale for the investigation of EGFR-TKI therapy in EGFR wild-type NSCLC. Stinchcombe et al argued that the practice pattern for EGFR wild-type NSCLC was platinum-based chemotherapy as first-line, immunotherapy as second-line, and single-agent chemotherapy as third-line therapy; nevertheless, only a small proportion of patients were eligible for fourth-line therapy and they should be enrolled in trials.^[19]^ The registered trials regarding anlotinib monotherapy for lung cancer were listed in Table 2. Moreover, there are also several trials of anlotinib plus chemotherapy or immune checkpoint inhibitors for the treatment of NSCLC, including ChiCTR1900028430 and ChiCTR1900028112.

**Table 2:**
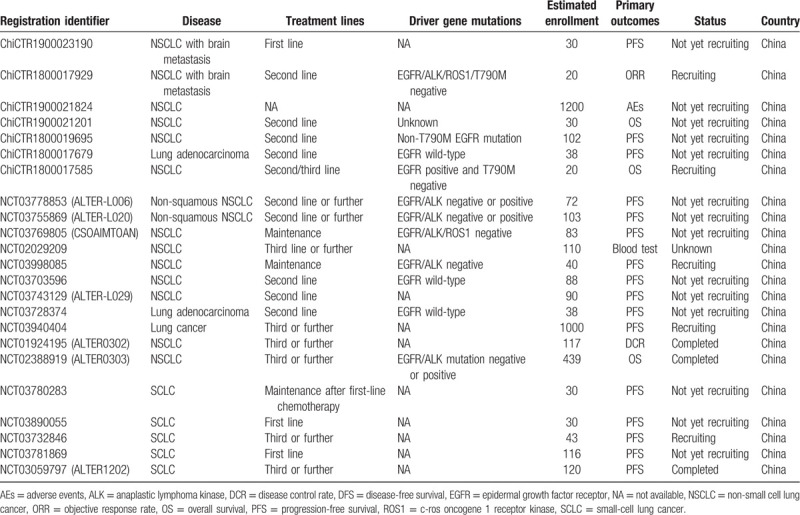
The registered trials of anlotinib monotherapy for the treatment of lung cancer.

In summary, anlotinib might be effective in EGFR wild-type lung adenocarcinoma after failure of chemotherapy. More evidence is needed to verify this occasional finding.

## Author contributions

**Conceptualization:** Lei Liu, Xiang Wang.

**Resources:** Miao Zhang.

**Writing – original draft:** Lei Liu, Wen-Bin Wu, Xiang Wang.

**Writing – review & editing:** Lei Liu, Miao Zhang.

## Abbreviations

Abbreviations: CT = computed tomography, MRI = magnetic resonance imaging, PFS = progression-free survival, OS = overall survival, VEGFR = vascular endothelial growth factor receptor, EGFR = epidermal growth factor receptor, TKI = tyrosine kinase inhibitor.
